# Luminal and mucosal-associated intestinal microbiota in patients with diarrhea-predominant irritable bowel syndrome

**DOI:** 10.1186/1757-4749-2-19

**Published:** 2010-12-09

**Authors:** Ian M Carroll, Young-Hyo Chang, Jiwon Park, R Balfour Sartor, Yehuda Ringel

**Affiliations:** 1 Division of Gastroenterology and Hepatology, Center for Gastrointestinal Biology and Disease, University of North Carolina at Chapel Hill, Chapel Hill, North Carolina, USA; 2 Korea Research Institute of Bioscience and Biotechnology (KRIBB), Dagjon, Korea

## Abstract

**Background:**

Recent studies have suggested a role for an altered intestinal microbiota in the pathophysiology of irritable bowel syndrome (IBS). However, no consensus has been reached regarding the association between specific enteric bacterial groups and IBS. The aim of this study was to investigate the fecal and mucosal-associated microbiota using two independent techniques in intestinal samples from diarrhea-predominant IBS (D-IBS) and healthy controls.

**Methods:**

Fecal and colonic mucosal biopsy samples were obtained from 10 D-IBS patients and 10 healthy controls. Colonic tissue was collected during a un-sedated un-prepped flexible sigmoidoscopy. Fecal and tissue samples were processed immediately upon collection for culture under aerobic and anaerobic conditions or frozen for further molecular analysis. DNA was extracted from all frozen samples and used to enumerate specific bacterial groups using quantitative real-time PCR (qPCR).

**Results:**

Culture analysis of intestinal samples demonstrated a significant reduction in the concentration of aerobic bacteria in fecal samples from D-IBS patients when compared to healthy controls (1.4 × 10^7 ^vs. 8.4 × 10^8 ^CFUs/g feces, *P *= 0.002). qPCR analysis demonstrated a significant 3.6 fold increase (*P *= 0.02) in concentrations of fecal *Lactobacillus *species between D-IBS patients and healthy controls.

**Conclusions:**

Our culture and molecular data indicate that quantitative differences exist in specific bacterial groups in the microbiota between D-IBS and healthy subjects.

## Background

Functional gastrointestinal disorders (FGID) are highly prevalent in Western countries with Irritable Bowel Syndrome (IBS) being the most common (affecting 10-20% of adults and adolescents)[1] and best studied condition. IBS is a heterogeneous disorder that can present as diarrhea-predominant IBS (D-IBS), constipation-predominant IBS (C-IBS), or mixed bowel habit IBS (M-IBS) subsets. Traditionally IBS has been considered a disorder that arises from an altered brain-gut axis that can be associated with gastrointestinal (GI) hypersensitivity and GI motor dysfunction[2,3]. Despite intensive research, the pathophysiology of this disorder is still unclear and no single etiological factor with a defined pathogenic mechanism has been identified. However, studies have implicated new theories that associate specific etiological factors in the pathogenesis of this disorder. These factors include alterations in the normal intestinal microbiota, genetic pre-determinants, pathogenic bacterial infection, food allergy, and an altered gut immune function and inflammation[4-6].

The intestinal microbiota is a complex community of bacteria, archaea, and eukarya. Indirect evidence that the intestinal microbiota plays a role in IBS comes from epidemiologic studies identifying acute GI infection (e.g., acute gastroenteritis) as a strong predictor for the development of IBS[7,8]. Additionally, antibiotic treatment of small intestinal bacterial overgrowth (SIBO) is associated with a resolution of IBS symptoms[9-11]. To further understand the role of the intestinal microbiota in the pathophysiology of IBS several studies have characterized this complex microbial community in IBS patients. Early studies using selective and non-selective culture techniques demonstrated differing viable levels of coliforms, *Lactobacillus*, *Bifidobacteria *and *Enterobacteriaceae *species in fecal samples from IBS patients[12,13]. More recent studies have used molecular methods to characterize an abnormality or dysbiosis in the intestinal microbiota of IBS subjects and demonstrated variations in the levels of the *Eubacterium*-*Clostridium coccoides *group and *Lactobacillus*, *Veillonella*, *Coprococcus*, *Collinsella*, *Coprobacillus *species in individuals with this disorder[14-21]. However, the majority of these studies used different, often mixed, patient populations and focused their analysis on one specific intestinal niche (fecal[12,16,17,20,21] or mucosal-associated[18] microbiota) with only one study investigating both niches[14]. As the luminal and mucosal-associated microbiota differs in composition[22], it is important to investigate and compare the microbiota of both of these niches.

In the present study we used two independent techniques to quantify and compare specific bacterial groups in fecal and colonic mucosal biopsy samples (collected in a manner that accurately maintained the composition of the microbiota) from patients with D-IBS and healthy controls.

## Results

### I. Study Population

A total of 20 subjects (10 D-IBS and 10 healthy controls) were investigated. All subjects provided fecal and colonic mucosal samples. The study population consisted of 70% females and had a mean age of 32 years. Demographics and body mass index (BMI) were similar in the two study groups (Table 1).

**Table 1:** Characteristics of D-IBS patients and Healthy Controls.

	D-IBS patients	Healthy Controls
**Number of subjects**	10	10
**Age (yr): mean (range)**	31.9 (23-50)	32.4 (21-54)
**Gender: F/M**	8/2	6/4
**BMI (kg/m**^**2**^**): mean (range)**	28.5 (23.1-40.6)	27.2 (19.9-36.3)

### II. Analysis of the fecal microbiota

The levels of aerobic bacteria in fecal samples from D-IBS patients were significantly lower compared to those from healthy controls (1.4 × 10^7 ^vs. 8.4 × 10^8 ^CFUs/g feces, *P *= 0.002) (Table 2). No significant differences were observed in the levels of anaerobic bacteria in fecal samples from D-IBS patients and healthy controls (6.24 × 10^9 ^vs. 3.12 × 10^9 ^CFUs/g feces, *P *= 0.3) (Table 2). Additionally, no significant differences between D-IBS patients and healthy controls were detected using selective media for *Bacteroides*, *Clostridium*, *Bifidobacteria*, *Lactobacillus *species and *Escherichia coli *concentrations in fecal samples (Table 2).

**Table 2:** Culture analysis of fecal and colonic mucosal samples from D-IBS patients and healthy controls.

	Aerobic*	Anaerobic*	*Clostridium *spp.*	*Bacteroides *spp.*	*Lactobacillus *spp.*	*Bifidobacterium *spp.*	E. coli*
**Fecal samples**:							
D-IBS patients	1.4 × 10^7^	6.2 × 10^9^	2.0 × 10^9^	8.6 × 10^8^	8.7 × 10^5^	1.1 × 10^9^	7.4 × 10^6^
Healthy controls	8.4 × 10^8^	3.1 × 10^9^	6.0 × 10^8^	3.5 × 10^8^	8.7 × 10^7^	6.4 × 10^8^	1.4 × 10^8^
*P *value^¥^	**0.002**	0.14	0.11	0.07	0.08	0.43	0.13
**Mucosal samples**:							
D-IBS patients	2.2 × 10^6^	9.3 × 10^6^	9.7 × 10^5^	4.6 × 10^6^	-^§^	1.3 × 10^6^	1.1 × 10^6^
Healthy controls	4.8 × 10^5^	6.5 × 10^6^	1.9 × 10^6^	1.4 × 10^6^	-^§^	5.9 × 10^5^	7.5 × 10^3^
*P *value^¥^	0.96	0.85	0.36	0.78	-	0.68	0.44

*Concentrations expressed in CFUs/g of sample.

^§ ^Below detection levels.

^¥ ^P value calculated using the non-parametric Mann-Whitney test.

**Figure 1:**
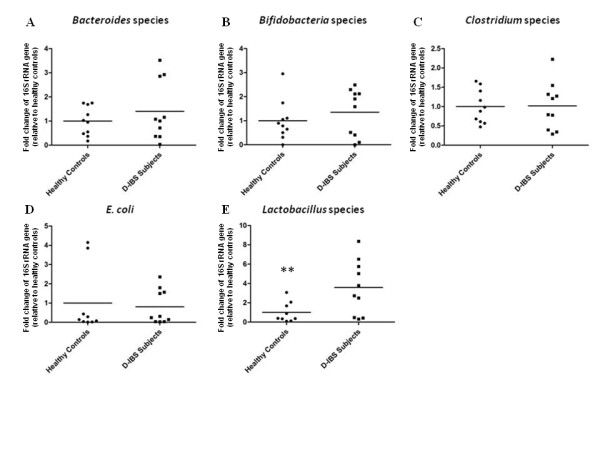
**Fold change in concentrations of (A) *Bacteroides *species, (B) *Bifidobacterium *species, (C) *Clostridium *species, (D) *E. coli*, and (E) *Lactobacillus *species in fecal samples from D-IBS patients and healthy controls using qPCR**. A significant increase in the levels of *Lactobacillus *species was detected in fecal samples from D-IBS patients (** *P *= 0.02).

**Figure 2:**
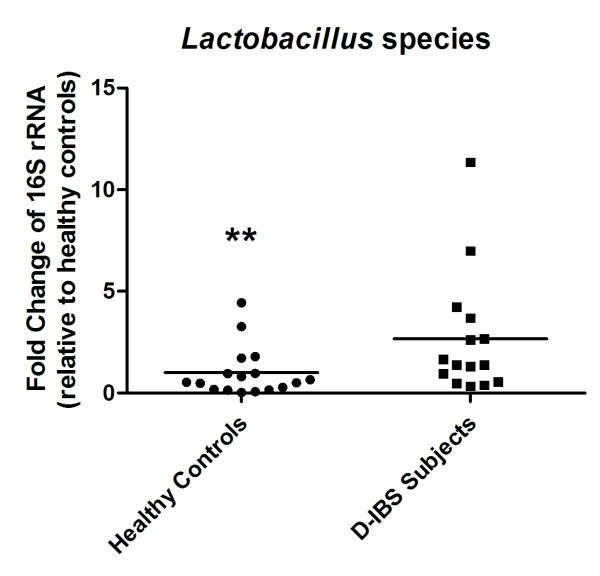
**Fold change in concentrations of *Lactobacillus *species in fecal samples from D-IBS patients and healthy controls using a larger sample number and alternative DNA isolation and qPCR methods**. A significant increase in the levels of *Lactobacillus *species was detected in fecal samples from D-IBS patients (** *P *= 0.02).

qPCR analysis detected the concentration of *Clostridium*, *Bacteroides*, *Bifidobacterium*, *Lactobacillus *species, and *E. coli *in all fecal DNA samples with the exception of 1 D-IBS samples that failed to amplify *Lactobacillus *species sequences. A significant 3.6 fold increase in the concentration of *Lactobacillus *species in fecal samples from D-IBS patients was observed when compared to healthy controls (HC = 10; D-IBS = 9, *P *= 0.02) (Figure 1E). No significant differences between groups were observed for concentrations of *Clostridium*, *Bacteroides*, *Bifidobacterium *species, and *E. coli *(Figure 1A-D). Investigation of bacterial groups using additional fecal samples from an on-going study (HC = 7, D-IBS = 6) using the same collection methods but alternative fecal DNA isolation and qPCR methods demonstrated similar results with a significant 2.7 fold increase in *Lactobacillus *species in D-IBS fecal samples compared to healthy controls (HC = 17; D-IBS = 15, *P *= 0.02) (Figure 2). Similarly, no significant differences between the groups in *Clostridium*, *Bacteroides*, *Bifidobacterium *species, and *E. coli *concentrations were detected between healthy controls and D-IBS patients.

### III. Analysis of the mucosal-associated intestinal microbiota

No significant differences were observed in the levels of aerobic or anaerobic bacteria in colonic mucosal samples between D-IBS patients and healthy controls (Table 2). Additionally, no significant differences between D-IBS and healthy controls were detected using culture on selective media for *Bacteroides*, *Clostridium*, *Bifidobacteria*, *Lactobacillus *species and *Escherichia coli *(Table 2).

**Figure 3:**
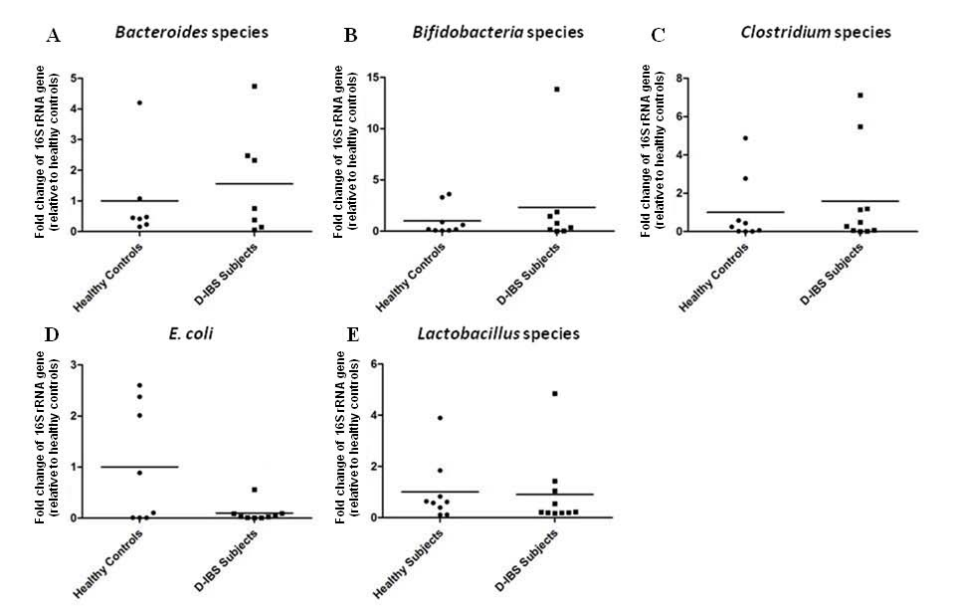
**Fold change in concentrations of (A) *Bacteroides *species, (B) *Bifidobacterium *species, (C) *Clostridium *species, (D) *E. coli*, and (E) *Lactobacillus *species in colonic mucosal samples from D-IBS patients and healthy controls using qPCR**.

qPCR analysis of colonic mucosal DNA did not reveal any significant differences between groups for *Clostridium*, *Bacteroides*, *Bifidobacterium*, and *Lactobacillus *species and *Escherichia coli *(Figure 3A-E).

### IV. Comparison of the fecal and mucosal-associated microbiota

Comparison of the fecal and mucosal-associated microbiota in healthy control and D-IBS groups revealed a significantly lower level of cultivable aerobic bacteria in colonic mucosal samples compared to fecal samples in both groups. However, the differences in cultivable aerobic bacteria observed between colonic mucosal and fecal samples from D-IBS patients was less obvious (Table 3). Both healthy control and D-IBS groups displayed a significant difference in cultivable anaerobes, *Bacteroides*, *Clostridium*, *Bifidobacteria *species and *E. coli *between mucosal and fecal niches (Table 3). These analyses could not be carried out on *Lactobacillus *species as the levels of these organisms were below detection limits in mucosal samples.

**Table 3:** Comparison of viable bacterial groups between fecal and colonic mucosal samples.

	Aerobic*	Anaerobic*	*Clostridium *spp.*	*Bacteroides *spp.*	*Lactobacillus *spp.*	*Bifidobacterium *spp.*	E. coli*
**Healthy controls**:							
Feces	8.4 × 10^8^	3.1 × 10^9^	6.0 × 10^8^	3.5 × 10^8^	8.7 × 10^7^	6.4 × 10^8^	1.4 × 10^8^
Mucosa	4.8 × 10^5^	6.5 × 10^6^	1.9 × 10^6^	1.4 × 10^6^	-^§^	5.9 × 10^5^	7.5 × 10^3^
*P *value^¥^	**0.0003**	**0.0001**	**0.02**	**0.004**	-	**0.04**	**0.019**
**D-IBS patients**:							
Feces	1.4 × 10^7^	6.2 × 10^9^	2.0 × 10^9^	8.6 × 10^8^	8.7 × 10^5^	1.1 × 10^9^	7.4 × 10^6^
Mucosa	2.2 × 10^6^	9.3 × 10^6^	9.7 × 10^5^	4.6 × 10^6^	-^§^	1.3 × 10^6^	1.1 × 10^6^
*P *value^¥^	**0.05**	**0.0001**	**0.0001**	**0.0001**	-	**0.0001**	**0.01**

*Concentrations expressed in CFUs/g of sample.

^§^Below detection levels.

^¥^*P *value calculated using the non-parametric Mann-Whitney test.

## Discussion

Recent studies have highlighted the importance of the intestinal microbiota in the well-being of the host. This diverse microbial community has been demonstrated as a critical factor for normal GI function[3,23,24]. As altered intestinal function is associated with IBS it is possible that an intestinal dysbiosis plays a role in the pathophysiology of the disorder. However, the investigation of the intestinal microbiota in IBS is difficult due to the heterogeneity of this condition, and the effects of an altered intestinal microbiota may not be consistent across all subtypes of IBS (D-IBS, C-IBS and M-IBS). In addition, the relative importance of the luminal versus the mucosal-associated niches in this disorder is not yet clear. Nevertheless, many of the studies that sought to characterize the intestinal microbiota in IBS investigated mixed populations of IBS patients[12,13,18], or focused on a single intestinal niche[12,13,15-21]. Thus, the aim of our study was to quantify and compare specific bacterial groups in both the luminal and mucosal-associated intestinal microbiota in a well-defined subgroup of patients with IBS and healthy controls. To achieve this we took the following measures; (a) we investigated a defined IBS subgroup population (D-IBS) to avoid variation in the microbiota compositions between different subtypes of IBS, (b) we used two independent techniques; culture on selective and non-selective media and qPCR, and (c) to avoid possible effects of time between delivery and analysis on the luminal microbiota, fresh fecal samples were collected from study subjects on site. These samples were immediately analyzed by culture or frozen for molecular analysis. To avoid the effect of bowel preparation on the mucosal-associated microbiota, colonic mucosal samples were collected from patients that did not receive a bowel preparation prior to the procedure.

The meticulous measures used in our study may explain some of the differences between our study results and previously reported studies investigating the intestinal microbiota in patients with IBS. For example, using non-selective culturing methods we found a decrease in concentrations of aerobic bacteria in D-IBS patients when compared to healthy controls. Additionally, using selective culture media we found no significant differences in concentrations of *Clostridium*, *Bacteroides*, *Bifidobacterium*, and *Lactobacillus *species or *E. coli *between D-IBS patients and healthy controls. These observations differ from those of previous studies that used culture techniques, where an increase in the total number of aerobic bacteria and a decrease in coliforms, *Lactobacillus *and *Bifidobacterium *species between IBS patients and healthy controls was reported[12,13,17]. However, these studies investigated a mixed population of IBS patients. In addition, one report investigated a population that included more than 50% of hospitalized patients[12], which may be a completely different population than the IBS population investigated in our study. It is appreciated that enumeration of specific bacterial groups using selective culture can be inaccurate and allow for organisms other than the species of interest to grow on a selective agar plate. Thus, the alternative and more accurate method of qPCR was used to enumerate different bacterial species in our samples.

Using qPCR our investigation demonstrated a significant increase in the levels of *Lactobacillus *species in fecal samples from D-IBS patients. As this finding was not *a priori *anticipated and contradict a previous study that reported a decrease in *Lactobacillus *species in D-IBS patients[16], we validated our results by reanalyzing our samples using alternate DNA extraction and qPCR methods on a larger set of fecal samples. Repeating the analysis using larger numbers and different methods yielded similar results of a significant increase in *Lactobacillus *species in the fecal samples from patients with D-IBS. Furthermore, our findings are supported by a study that demonstrated an increase in *Lactobacillus *species in IBS subjects (a mixed subtype population) using selective culturing techniques[19]. The differing reported results may be a reflection of the different ways of collecting samples as well as the alternate fecal DNA extraction and qPCR methods used. In our study we initially used a method that solely relied upon a high temperature to lyse bacterial cells. As this method may have a bias for bacteria with weaker cell walls (e.g. Gram negative bacteria) we utilized an independent method that ensures complete lysis of bacterial cells with enzymatic (lysozyme and proteinase K), chemical (sodium dodecyl sulfate), and physical disruption (bead-beating) steps. Additionally, we incorporated a universal bacterial primer set in our qPCR assay. The advantage of using this approach is that the bacterial group under investigation is determined as a percentage of total 16S rRNA genes in a sample. In addition, the differences in the reported results may relate to the differences in the study population since the reported decrease in *Lactobacillus species *was observed when comparing D-IBS and C-IBS, but not compared to healthy controls[16].

Our analysis also compared different bacterial groups harbored within fecal and mucosal niches of the intestine. We observed that in healthy individuals and D-IBS subjects the total number of cultivatable aerobic bacteria significantly differed between the microbiota located at these two intestinal sites. However, in D-IBS patients the difference in the levels of aerobic bacteria found between fecal and mucosal niches was less evident. Interestingly, an opposite trend was observed in cultivable *Bacteroides *species, where a significant decrease in the levels of this bacterial group was more obvious between mucosal and fecal niches in D-IBS patients than healthy controls. The remaining bacterial groups investigated showed similar trends between mucosal and fecal niches in both D-IBS patients and healthy controls. At this point it is not known which anaerobic bacteria or *Bacteroides *species are associated with these differences in intestinal niches between healthy individuals and D-IBS patients. However, these observations warrant further examination.

To date two studies have investigated the microbiota of luminal and mucosal niches within the intestine of IBS subjects[14,25]. However, the first of these studies did not collect mucosal samples from healthy controls[14]. In the second study, fecal and duodenal mucosa brush samples were collected from IBS subjects and healthy controls[25]. Our study differed to these previous reports as we collected fecal and colonic mucosal samples from D-IBS subjects and healthy controls. Together our study and these previous reports highlight the importance of investigating both luminal and mucosal niches in the intestine of IBS subjects and healthy controls.

## Conclusions

Our data demonstrate differences in both the luminal and mucosal-associated intestinal microbiota between patients with D-IBS and healthy controls. These data suggest that both fecal and mucosal intestinal niches may independently have an important association with D-IBS. The clinical relevance of these observations still need to be addressed as it is difficult to establish whether differences in the intestinal microbiota between D-IBS patients and healthy controls are a cause of the disorder or an effect of the altered intestinal function or luminal environment in these patients. It is also appreciated that this study focused on a limited number of clinically relevant bacterial species in a small sample of patients and does not provide a generalized view of the diverse intestinal microbiota. However, our study presents interesting new findings that substantiates further in depth investigation of the fecal and mucosal-associated intestinal microbiota in IBS and sub-types of IBS using methods that characterize the composition of the intestinal microbiota in greater details.

## Methods

### Study Population

We studied 10 patients that met the Rome III criteria for D-IBS and 10 healthy controls[1]. Subjects were recruited from the Chapel Hill general population by advertising and from the University of North Carolina (UNC) at Chapel Hill outpatient clinics.

Inclusion criteria comprised of subjects at least 18 years of age and of any gender, race, or ethnicity. All subjects were evaluated by a physician to exclude an alternative diagnosis to IBS. D-IBS subjects had active GI symptoms at the time of sample collection. Healthy controls had no significant recurring GI symptoms. Subjects with a history of GI tract surgery other than appendectomy or cholecystectomy, a history of inflammatory bowel diseases (IBD), celiac disease, lactose malabsorption, or any other diagnosis that could explain chronic or recurring bowel symptoms were excluded from the study. In addition, participants were excluded if they had a history of antibiotic treatment or intentional probiotic consumption two months prior to the beginning of the study.

The extra subjects used to validate qPCR analysis (HC = 7, D-IBS = 6) were recruited in the same manner. The study was approved by the UNC Internal Review Board (IRB) and all subjects signed a consent form prior to participation in the study.

### Sample Collection and Preparation

Fresh stool samples were collected from all 20 subjects on site during the study visit at UNC. Each fecal sample was immediately placed in an anaerobic pouch system (AnaeroPack^® ^System, Misubishi Gas Chemical America, Inc.) and transferred on ice to the laboratory. In the laboratory, each stool sample was homogenized and divided into aliquots. Samples were immediately used for culture of viable bacteria and the remaining aliquots were stored at -80°C for DNA extraction and qPCR analysis. The extra 13 subjects included to validate our analyses (HC = 7, D-IBS = 6) provided a fecal sample which processed in the same manner for DNA extraction and qPCR analysis.

Three colonic mucosal biopsies were collected from each subject during an un-sedated flexible sigmoidoscopy. To avoid possible effects of colonic preparation on the intestinal microbiota all the procedures were carried out on un-prepped colons. Colonic mucosal biopsies were taken from the distal colon just above the rectosigmoid junction using cold forceps. Once removed from the colon each biopsy was washed in 1 ml of sterile PBS to remove non-adherent bacteria. The biopsies were then weighed and used immediately for culture of viable bacteria while other samples were flash-frozen in liquid nitrogen for further DNA extraction and qPCR analysis.

### Culture of Fecal and Mucosal Microorganisms

An aliquot of a fresh fecal sample or colonic biopsy was aseptically added to sterile phosphate buffer saline (PBS) to obtain a final volume of 1 ml. Fecal samples were vortexed until a homogenous suspension was obtained and colonic biopsies were vortexed for 2 min to ensure the release of all adherent bacteria. The mixture was then serially diluted and spread onto appropriate selective and non-selective agar plates for the detection of specific bacterial groups by aerobic and anaerobic culture. The concentrations of each bacterial group were expressed as the number of colony forming units (CFUs) per gram of sample. The following types of micro-organisms were enumerated:

#### Total Bacterial Counts

Total aerobic and anaerobic numbers were determined by culturing diluted samples on Brain Heart Infusion (BHI) agar plates (Difico™, Franklin Lakes, NJ) supplemented with L-cystine (0.05%) and hemin (5 mg L^-1^). Agar plates were incubated aerobically at 37°C for 24 hr to enumerate total aerobic bacteria or anaerobically (10% H_2_, 80% N_2_, and 10% CO_2_) for 48 hr to enumerate total anaerobic bacteria. All colonies encompassing different morphologies were counted on these plates.

#### *Bifidobacteria *species

Columbia agar base plates (Difico™, Franklin Lakes, NJ) supplemented with L-cystine (0.05%), hemin (5 mg L^-1^), horse blood (5%), and a bile salt solution (bile salts: Sodium propionate - 4.5 g L^-1^, paromonycin sulphate - 15 g L^-1^, Neomycin sulfate - 60 g L^-1^and Lithium chloride - 900 mg g L^-1^) were used to culture and enumerate *Bifidobacteria *species. All incubations were carried out anaerobically at 37°C.

#### *Lactobacillus *species

Man, Rogosa, and Sharpe (MRS) agar plates (Difico™, Franklin Lakes, NJ) supplemented with L-cystine (0.05%) and hemin (5 mg L^-1^) were used to enumerate *Lactobacillus *species. All incubations were carried out anaerobically at 37°C.

#### *Escherichia coli*

M^c^Conkey agar (Difico™, Franklin Lakes, NJ) was used to enumerate *E. coli*. All incubations were carried out aerobically at 37°C.

#### *Clostridium *species

M^c^Clung Toabe agar plates (Difico™, Franklin Lakes, NJ) were used to enumerate total *Clostridium *species. All incubations were carried out anaerobically at 37°C.

#### *Bacteroides *species

Bacteroides Bile Esculin agar plates (Difico™, Franklin Lakes, NJ) were used to enumerate *Bacteroides *species. All plates were incubated anaerobically at 37°C.

### Extraction of Fecal DNA

#### Fecal Samples

Fecal DNA was extracted using the QIAamp^® ^DNA stool mini kit (Qiagen, Valencia, CA). Briefly, 200 mg was taken from each frozen stool sample and placed immediately into ASL buffer. Each fecal sample was homogenized by vortexing. The mixture was then heated to 95°C for 5 min to obtain bacterial lysis. Fecal DNA was further extracted and purified as per the manufacturer's instructions. DNA concentrations were determined using a NanoDrop™ (Thermo Scientific, Wilmington, DE). All fecal DNA samples were adjusted to equal concentrations for subsequent qPCR analysis.

To validate our molecular observations an alternate DNA extraction method was applied to all fecal samples with the addition of 13 extra stool specimens (HC = 7, D-IBS = 6) from an ongoing study (total sample number, HC = 17, D-IBS = 16). This method used more rigorous steps to lyse bacterial cell walls. Briefly, DNA from fecal samples was extracted using a phenol/chloroform extraction method combined with physical disruption of bacterial cells and a DNA clean-up kit (Qiagen DNeasy^® ^Blood and Tissue extraction kit). 100 mg of frozen feces was suspended in 750 μl of sterile bacterial lysis buffer (200 mM NaCl, 100 mM Tris [pH 8.0], 20 mM EDTA, 20 mg/ml lysozyme [Sigma-Aldrich, St. Louis, MO]) and incubated at 37°C for 30 min. Next, 40 μl of proteinase K (20 mg/ml) and 85 μl of 10% SDS was added to the mixture and incubated at 65°C for 30 min. 300 mg of 0.1 mm zirconium beads (BioSpec Products, Bartlesville, OK) was then added and the mixture and homogenized in a bead beater (BioSpec Products, Bartlesville, OK) for 2 min. The homogenized mixture was cooled on ice and then centrifuged at 14,000 rpm for 5 min. The supernatant was transferred to a new 1.5 ml microfuge tube and fecal DNA was further extracted by phenol/chloroform/iso-amyl alcohol (25:24:1) and then chloroform/iso-amyl alcohol (24:1). The supernatant after extraction was precipitated by absolute ethanol at -20°C for 1 hour. The precipitated DNA was suspended in DNase free H2O and then cleaned using the DNeasy^® ^Blood and Tissue extraction kit (Qiagen) from step 3 as per the manufacturer's instructions.

#### Mucosal Samples

DNA from colonic mucosal biopsies was extracted using the Qiagen Allprep DNA/RNA kit™ (Qiagen, Valencia, CA) with the addition of a lysozyme and bead-beating step. Briefly, each biopsy was incubated in 300 μl of a lysozyme solution (30 mg/ml) for 30 min at 37°C. Next, 600 μl of RLT buffer (containing β-mercaptoethanol) and 300 mg of 0.1 mm zirconium beads (BioSpec Products, Bartlesville, OK) were added and the solution was homogenized in a bead beater (BioSpec Products, Bartlesville, OK) for 2 min. The solution was centrifuged for 5 min at 14,000 rpm and DNA was further extracted from the supernatant as per the manufacturer's instructions.

### Quantitative PCR

Quantitative PCR (qPCR) was performed using the QuantiTect SYBR^® ^Green PCR kit (Qiagen, Valencia, CA) with primers that amplify the genes encoding 16S rRNA from specific bacterial groups. The primers used to amplify specific groups of are listed in Table 4. qPCR assays were conducted in 96-well plates on a real-time MX 3000*P *thermocycler (Stratagene, La Jolla, CA). Each PCR was carried out in a final volume of 25 μl and contained the following: 1 × SYBR green qPCR Master Mix (Qiagen), 0.5 μM of each primer and 50 ng of purified fecal or colonic mucosal DNA. PCR conditions were as follows: 15 min at 95°C, followed by 40 cycles of 95°C for 1 min, 30 s at the appropriate annealing temperature (Table 4), and 72°C for 1 min. Each plate included duplicate reactions per DNA sample and the appropriate set of standards. qPCR standards were generated by PCR amplifying and cloning the target 16S rRNA genes from an appropriate positive control strain. Melting curve analysis of the PCR products was conducted following each assay to confirm that the fluorescence signal originated from specific PCR products and not from primer-dimers or other artifacts. All qPCR plates included a 'no template' negative control for each primer set. The concentrations of each bacterial group in D-IBS patients were expressed as a 'fold change' with respect to the control group. All microbiology analyses of fecal and colonic mucosal samples were performed blindly, without knowledge of the subjects' clinical data.

**Table 4:** Quantitative polymerase chain reaction (qPCR) primes used in this study to enumerate specific bacterial species.

Bacterial species		Primer Sequence 5'-3'	Annealing temperature (°C)	PCR Product size (bp)
*Bacteroides *spp.^a^	F- R-	ATAGCCTTTCGAAAGRAAGAT CCAGTATCAACTGCAATTTTA	50	495
*Clostridium *spp.^b^	F- R-	CGGTACCTGACTAAGAAGC AGTTTYATTCTTGCGAACG	50	429
*Escherichia coli*^c^	F- R-	GTTAATACCTTTGCTCATTGA ACCAGGGTATCTAATCCTGTT	52	340
*Bifidobacterium *spp.^d^	F- R-	GGGTGGTAATGCCGGATG TAAGCGATGGACTTTCACACC	55	442
*Lactobacillus *spp.^e^	F- R-	AGCAGTAGGGAATCTTCCA CACCGCTACACATGGAG	50	341

^a^primers from Matsuki *et al*.,[26]

^b^primers from Rinttila *et al*.,[27]

^c^primers from Malinen *et al*.,[28]

^d^primers from Bartoch *et al*.,[29]

^e^primers from Maeda *et al*.,[30]

To validate our qPCR findings an alternate qPCR method was applied to our larger number of fecal DNA samples (HC = 17, D-IBS = 16). Briefly, qPCR was performed using ABI SYBR^® ^Green PCR Mastermix (Applied Biosystems, Carlsbad, CA) with primers that amplify the genes encoding 16S rRNA from *Lactobacillus *species (Table 4) or all bacteria (total bacterial 16S rRNA in each sample was determined using universal 16S rRNA primers; forward, 5'-GTGSTGCAYGGYTGTCGTCA-3'; reverse, 5'-ACGTCRTCCMCACCTTCCTC-3') in fecal DNA from D-IBS patients and healthy controls. qPCR assays were conducted in 96-well plates on an Eppendorf Realplex^2 ^mastercycler thermocycler (Eppendorf, Hauppauge, NY). Each PCR was carried out in a final volume of 12 μl and contained the following: 1 × SYBR green mastermix, 0.5 μM of each primer and approximately 50 ng of purified fecal DNA. PCR conditions were as follows: 15 min at 95°C, followed by 40 cycles of 95°C for 15 s, 30 s at the appropriate annealing temperature (Table 4), and 72°C for 45 s. Each plate included duplicate reactions per DNA sample and the appropriate set of standards. The concentration of *Lactobacillus *species were expressed as a percentage of total 16S rRNA sequences in a given sample. Analysis of melting curves confirmed that the fluorescence signal originated from specific PCR products and not from primer-dimers or other artifacts. All qPCR plates included a 'no template' negative control for each primer set. The concentrations of each bacterial group in D-IBS patients were expressed as a 'fold change' with respect to the control group.

### Statistical Analysis

For culture analysis the total number of CFUs per gram of feces for aerobic, anaerobic, and each specific bacterial species investigated was determined for each sample. Mean total aerobic, anaerobic and species specific CFUs were compared between D-IBS patients and healthy controls using the non-parametric Mann-Whitney test. Similarly, for qPCR assays the concentration of each bacterium/bacterial group was determined for each sample. Mean number of 16S rRNA sequences per μg of sample DNA was compared between D-IBS patients and healthy controls using the non-parametric Mann-Whitney test. Statistical analysis was carried out using GraphPad software (v4.0a; Prism, San Diego, CA).

## List of Abbreviations

IBS: irritable bowel syndrome; D/C/M-IBS: diarrhea/constipation/mixed bowel habit-predominant IBS; HC: healthy controls; CFU: colony forming units; qPCR: quantitative real-time PCR; FGID: functional gastrointestinal disorders; SIBO: small intestinal bacterial overgrowth.

## Competing interests

The authors declare that they have no competing interests.

## Authors' contributions

IMC - molecular techniques, statistical analysis, and scribe of manuscript.

YHC - collection of biological samples and culture techniques.

JP - culture techniques.

RBS - study design.

YR - study design, study execution, recruitment of subjects, IRB application scribe, and recipient of funding for study.

All authors read and approved the final draft.

## Acknowledgements and Funding

The authors would like to acknowledge Sarah Van Heusen and Sarah Yeskel for their valuable contributions to this study. This study was funded by a DK067674 seed grant from the UNC Center for Functional GI Disorders awarded to YR.
